# Interventions on soy isoflavone molecules to improve their therapeutic potential for prostate cancer treatment

**DOI:** 10.17179/excli2022-5130

**Published:** 2022-07-01

**Authors:** Vladimir Ajdžanovic, Branka Šošic-Jurjevic, Vesna Vodnik, Branko Filipovic

**Affiliations:** 1 Department of Cytology, Institute for Biological Research "Siniša Stankovic" - National Institute of the Republic of Serbia, University of Belgrade, Belgrade, Serbia; 2 Department of Radiation Chemistry and Physics "GAMMA", "Vinca" Institute of Nuclear Sciences - National Institute of the Republic of Serbia, University of Belgrade, Belgrade, Serbia

## ⁯

Recent global data suggest that prostate cancer represents the second most frequent cancer and the fifth leading cause of cancer deaths among men, with almost 1.4 million new cases and 375000 deaths in 2020 (Sung et al., 2021[7]). Well-established risk factors for prostate cancer are family history, race and hereditary syndromes, while a limited number of modifiable risk factors may determine developing prostate cancer, so little evidence exists in terms of the disease prevention (Gandaglia et al., 2021[4]). Soy isoflavones (genistein and daidzein are the best known representatives) are prominent as promising compounds in the prevention of prostate cancer, with observable but discrete effects (and some limitations) in cancer treatment, especially in its metastatic phase (Ajdžanović et al., 2019[1]). In line with this, mechanistic studies indicate that soy isoflavones may affect various pathologically active signaling pathways in prostate cancer cells, downregulate the cancer cell androgen receptors, decrease the expression of prostate-specific antigen and matrix metalloproteinase, reverse prostate cancer cell epithelial to mesenchymal transition, contribute to epigenetic changes associated with the fate of cancer cells and suppress the angiogenesis that follows prostate cancer growth (Ajdžanović et al., 2019[1]). The limiting factor for the clinical use of soy isoflavones is their low bioavailability, due to poor water solubility, rapid metabolism and excretion (Tang et al., 2019[8]). Advances in chemo-, immuno- and radio-therapy dictate the fact that plant-derived compounds (formulated as dietary supplements/nutraceuticals) are not the first-line treatment for metastatic prostate cancer. However, soy isoflavone therapeutic ranking is rising as evidence accumulates regarding the effectiveness of combining therapeutic approaches with soy isoflavone participation (Ajdžanović et al., 2019[1]). Given all the above, it can be said that there is a need for further tuning of prostate cancer treatment that involves soy isoflavones.

Enhancement of the therapeutic potency of soy isoflavones and upgrade of their pharmacokinetic profiles/bioavailability may be achieved by means of different interventions on these isoflavones, either by chemical engineering based on their structure or by designing efficient soy isoflavone delivery systems (Vodnik et al., 2021[9]; Xiong et al., 2015[10]). At the practical level, there are three modalities of such interventions: chemical modifications, synthesis of analogues and coupling with nanoparticles. Chemical modifications, including the possibility of increasing soy isoflavone molecules' lipophilicities through complexation with transient metal cations, to give modified compounds with desirable inputs on prostate cancer cell signaling machinery (Ajdžanović et al., 2015[2]), still appear far from realization. Some practical experience is available with the other two modalities of interventions using soy isoflavone molecules, so their effects on prostate cancer cells and tumors is the main subject of elaboration in the following table (Table 1(Tab. 1); References in Table 1: Barve et al., 2006[3]; Jackson et al., 2019[5]; Stolarczyk et al., 2021[6]; Vodnik et al., 2021[9]; Xiong et al., 2015[10]).

According to the data summarized in Table 1(Tab. 1) (especially given the findings shown in bold), some progress has been made in improving the therapeutic potential of soy isoflavone molecules for the treatment of prostate cancer. at higher doses, genistein analogues have generally more pronounced antiproliferative effects on metastatic prostate cancer cells *in vitro* than daidzein, while thiogenistein shows even better performance than genistein within the same context. Nanosuspension of genistein, in combination with radiation, has been shown to be effective in suppressing prostate tumor growth. Genistein-gold nanoparticle conjugates possess lower toxicity than genistein against non-malignant human cells. Verification of these results requires further intensive research, primarily *in vivo* in animal models, and subsequently at the pre-clinical and clinical levels. Special attention should be paid to defining the optimal dosage range of modified soy isoflavone molecules in the treatment of prostate cancer, to achieve the best therapeutic results.

## Declaration

### Acknowledgments

This work was supported by the Ministry of Education, Science and Technological Development of the Republic of Serbia, via direct financing of our Institutes (contract numbers: 451-03-68/2022-14/200007 and 451-03-68/2022-14/200017). We are grateful to Prof. Dr. Steve Quarrie, an English language professional, for his help in proofreading the manuscript.

### Conflict of interest

The authors declare that they have no conflict of interest.

## Figures and Tables

**Table 1:**
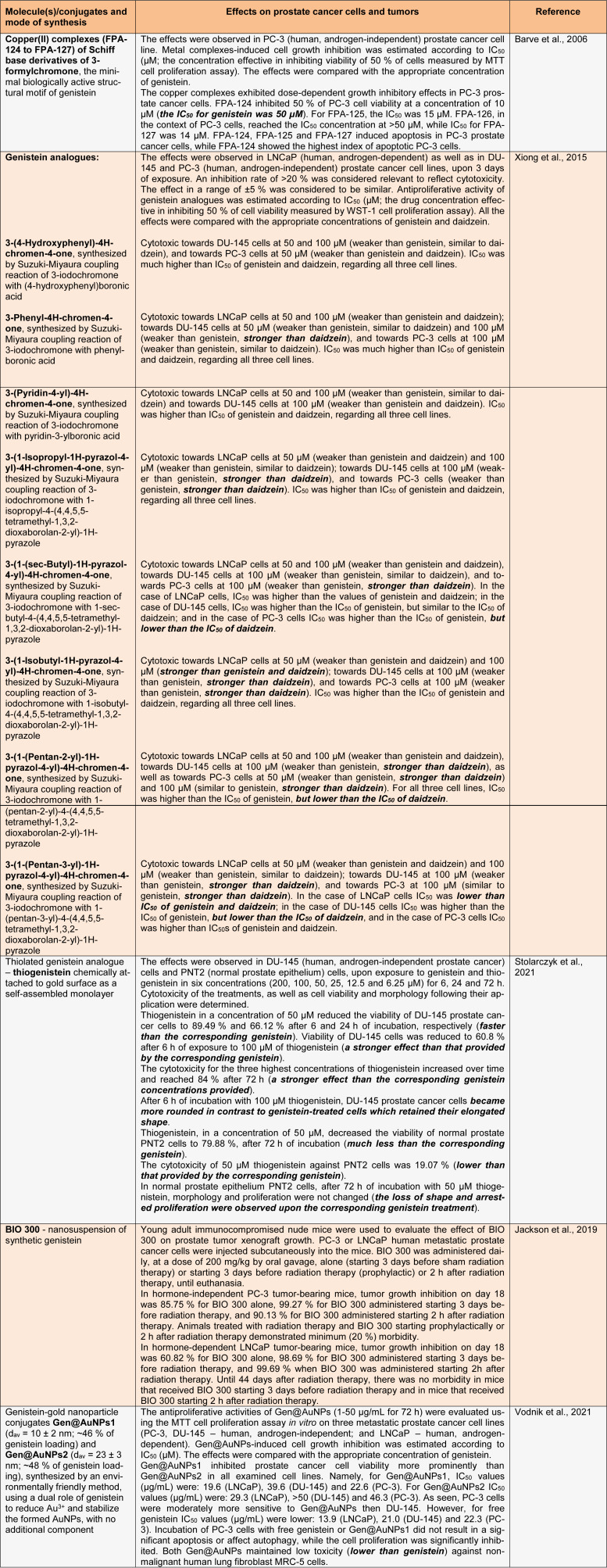
A review of interventions on soy isoflavone molecules that have been performed to improve their therapeutic potential for prostate cancer treatment
